# Enhancing Thermoelectrical Properties of Silver-Nanowire-Embedded Heatable Textiles via Sputter-Mediated Nanowire Structural Modulation

**DOI:** 10.3390/ma17225514

**Published:** 2024-11-12

**Authors:** Chankyoung Lee, Jaewoo Park, Dooho Choi

**Affiliations:** Department of Semiconductor Engineering, Gachon University, Seongnam 13120, Republic of Korea; tozion1@gachon.ac.kr (C.L.); pjpj1209@gachon.ac.kr (J.P.)

**Keywords:** Ag nanowires, sputtering, heatable textiles, Joule heating

## Abstract

This study addresses the fabrication of flexible, heatable fabrics via the integration of globally interconnected silver nanowires (Ag NWs) with sputter-deposited silver atoms. Conventional heatable fabrics, which utilize macroscale or nanoscale conductive wires, often face challenges in balancing flexibility, comfort, and structural durability. The proposed method leverages the advantages of nanoscale metallic wires and vacuum-based sputtering, maintaining fabric flexibility while enhancing heating efficiency. The fabrication process involves dip-coating polyester fabric with Ag NWs, followed by sputter deposition to modulate the nanowire morphology, thereby improving key electrical properties such as wire resistance and contact resistance between wires. The experimental results demonstrate that sputter-deposited Ag NW fabrics exhibit significantly enhanced heating capability compared to undeposited, otherwise identical counterparts. Further, the fabrics maintain their heating characteristics under repeated mechanical bending and prolonged electrical stress, highlighting their potential for use in wearable electronic applications. This approach offers a promising solution to the limitations of current heatable textile technologies, providing a pathway for the development of comfortable, efficient, and durable heatable fabrics.

## 1. Introduction

Recently, extensive research has been conducted on smart textiles incorporating nanomaterials and nanotechnologies, among which battery-powered heatable fabrics have garnered much attention [1,2,3,4,5,6]. The widely accepted approach for creating heatable fabrics primarily involves the integration of either macroscale or nanoscale conductive wires [7,8,9,10,11,12]. While macroscale conductive wires, possessing low electrical resistance, are efficient heat generators, they tend to increase fabric stiffness, which compromises flexibility and comfort [13,14]. This stiffness can also lead to permanent damage under repeated mechanical deformations such as bending and twisting. Conversely, nanoscale wires offer improved flexibility and comfort but are susceptible to structural degradations when subjected to concurrent thermal and electrical stresses. Further, achieving a spatially uniform distribution of metallic nanowires (NWs) poses a challenge, often resulting in a non-uniform thermal profile and accelerated structural deterioration due to localized hot spots [15,16,17,18]. Carbon-based network heating elements such as carbon fiber [19], graphene [20], and carbon nanotubes [21] have also been widely studied for use in heatable fabrics. However, these heating elements exhibit considerably higher electrical resistance [22,23], which requires substantially greater operating voltages, posing practical limitations. Vacuum-based deposition methods such as sputtering are superior in forming uniform heating elements in the form of thin films suitable for flat substrates. However, these approaches, without excessive deposition, may not produce globally continuous conducting layers on fabrics, which typically consist of isolated yarns.

In this study, we propose a method to overcome the conventional limitations of NW-based and thin-film-based heatable fabrics by combining globally interconnected Ag NWs with sputter-deposited Ag atoms to enhance the heating characteristics. Sputter deposition can structurally alter the nanowire shape (i.e., NW diameter), thereby modulating the NW resistance and promoting tighter contact between NWs to reduce contact resistance. This combination preserves the porous fabric structure, thus offering comfort, breathability, and moisture absorption [24,25,26,27]. We employed repeated dip-coating in an Ag NW solution followed by a drying process to produce a globally interconnected structure on polyester [28,29,30]. Subsequently, sputter-deposited Ag atoms were used to modulate the heating characteristics of the NWs. The resulting structure exhibited superior thermal response and stability compared to pristine Ag NW heating elements without sputtering. Furthermore, the combined structure demonstrated excellent flexibility and durability, underscoring the suitability of our approach for heatable textile applications. While we evaluated the applicability of our approach using polyester composed of separated yarns, the results of this study can also be extended to other fabric types, such as cotton with a similar yarn structure [31,32].

## 2. Experiment

**Figure 1:**
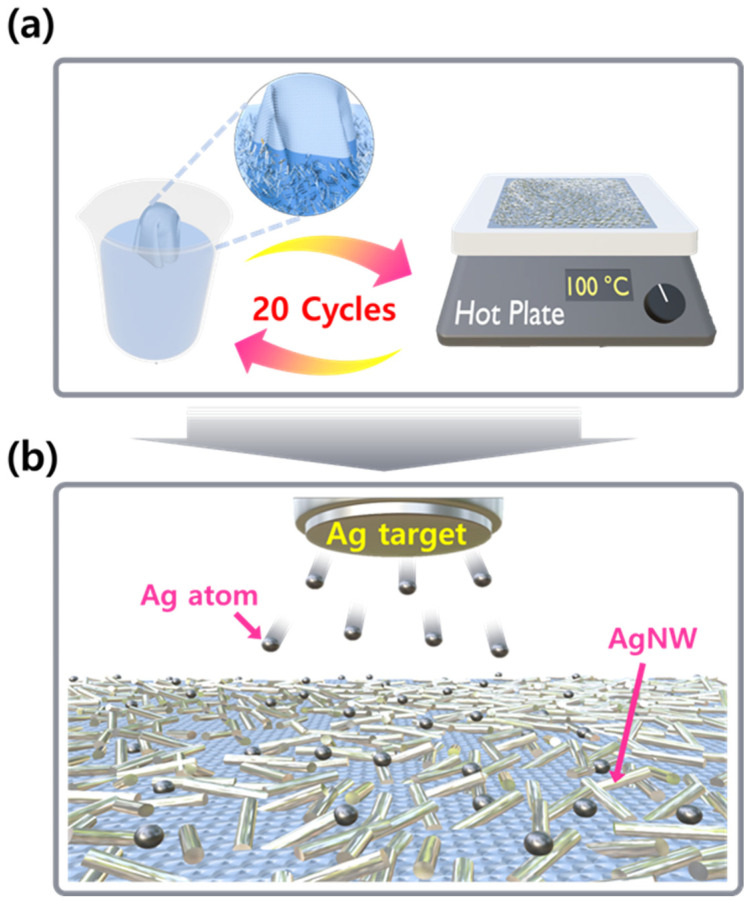
(**a**) A schematic representation of the transfer process of Ag NWs onto the polyester fabric surface through the dipping and drying process. (**b**) A schematic illustrating the sputtering process for Ag deposition onto the Ag-NW-embedded fabric.

Ag NWs with an average length of 30 μm and a diameter of 30 nm were dispersed onto polyester fabric through a dipping and drying process. Ag NWs were suspended in isopropyl alcohol (IPA) at a concentration of 0.05 g/L, and the fabric underwent 15 cycles of immersion and drying at 100 °C for 1 min. The formation of the Ag NW network on the fabric during this process is illustrated schematically in Figure 1a. To further modulate the Ag NW morphology and the corresponding conductance of the Ag NW networks, Ag atoms were deposited onto the fabric by radio frequency (RF) magnetron sputtering (Figure 1b). This deposition was conducted using a 3-inch diameter Ag target (99.999 wt%) with a sputtering power of 50 W. Prior to deposition, the chamber base pressure was maintained below 2.0 × 10^−6^ torr, and Ar gas (99.9999%) was introduced to raise the pressure to 4 × 10^−2^ torr before commencing the sputtering process. To promote spatial uniformity, the fabric substrates were rotated at 15 rpm. To measure the Ag deposition rate, partially covered masks were formed on glass substrates prior to Ag deposition by dropping and solidifying the photoresist. After deposition, the photoresist was removed by acetone in an ultrasonic bath to create steps, and the step heights (film thicknesses) were measured using a surface profiler (D-100, KLA Tencor, Milpitas, CA, USA). For reliable measurement, the Ag layer thicknesses were kept above 300 nm, and the reference deposition rate for Ag (0.27 nm/s) was derived from these measurements. The alterations in Ag NW morphology with the increasing duration of Ag deposition were observed using a field emission scanning electron microscope (SEM, JSM-800, JEOL Ltd., Akishma, Japan).

The polyester fabric (WW-300, KM Corp., Seoul, South Korea) used in this study has a mass per unit area of 168 ± 17 g/m^2^, a tensile strength of 150 kgf/cm^2^, and a total thickness of 580 ± 20 μm. The as-purchased 3 cm × 3 cm polyester fabric containing the Ag NW network was sputter-coated with Ag at varying equivalent layer thicknesses (ELTs) of 5, 10, 20, 30, 40, 50, and 60 nm. For the assessment of Joule heating performance, 200 nm thick and 5 mm wide Ag electrodes were deposited along the opposite parallel sides of the fabric using an electron-beam evaporator. Copper tape was then attached for electrical contact by applying uniform manual pressure until no noticeable local thermal variations were observed. The Ag electrode effectively promoted adhesion between the fabric and copper tape. The resistance of the heatable fabrics was measured immediately after fabrication using a True-RMS multimeter (Fluke116, Flusk Corp., Everett, WA, USA). Joule heating tests were conducted using an external power supply (EPS-3305, EZT, Seoul, South Korea), and the surface temperature of the heated textiles in response to the applied voltage was recorded with an infrared camera (PTI-120, Fluke Corp., Everett, WA, USA).

## 3. Result and Discussion

**Figure 2:**
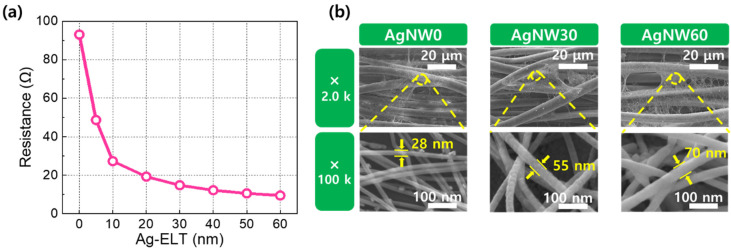
(**a**) The resistance of Ag-NW-embedded fabrics as a function of Ag-ELT. (**b**) SEM micrographs showing the morphologies of AgNW0, AgNW30, and AgNW60, respectively. The average Ag NW diameters are included in the images.

Figure 2a shows the resistance of Ag-NW-embedded fabric as a function of Ag-ELT, which exhibits a continuous decrease in resistance as the Ag-ELT increases. The SEM images in Figure 2b visually confirm that increasing the Ag-ELT (0, 30, and 60 nm) on the Ag-NW-embedded fabric (AgNW0, AgNW30, and AgNW60) modulates the NW morphology, contributing to the resistance reduction. The Ag NW widths for AgNW0, AgNW30, and AgNW60 are 28 ± 1.6, 55 ± 2.2, and 70 ± 2.8 nm, respectively, with 95% confidence intervals. These results clearly demonstrate that the NW dimensions increase with extended sputtering duration. It is notable that a more pronounced resistance reduction is observed in the low Ag-ELT regime. For example, the Ag-ELT of 10 nm results in a 60% reduction in resistance relative to the undeposited case, whereas a 90% reduction is observed between 50 and 60 nm of Ag-ELT. This non-uniform resistance change can be attributed to two factors. First, the sputter deposition promotes tighter contacts between the Ag NWs. Second, the severity of electron scattering at the NW surfaces is mitigated with decreasing NW dimensions [33,34,35,36,37]. This effect is particularly significant given the relatively long electron mean free path of 54 nm for Ag. As the NW diameter approaches or falls below the mean free path, the frequency of electron collisions increases, leading to a greater resistance reduction. These findings indicate that sputtering is an efficient method for modulating the Ag NW structural dimensions and, consequently, their electrical properties. It is also noteworthy that prolonged sputtering, corresponding to an Ag-ELT of 200 nm, on fabric without an embedded Ag NW network does not yield measurable resistance (i.e., exceeding the instrumental limit of 40 MΩ), as sputtering alone fails to electrically connect adjacent isolated yarns. Given the high contact resistance between undeposited Ag NWs and the challenges of achieving a uniform distribution of high-density metallic nanowires, combining a globally interconnected Ag NW network with sputtering to modulate their structural and electrical properties offers an efficient strategy for fabricating heatable fabrics.

**Figure 3:**
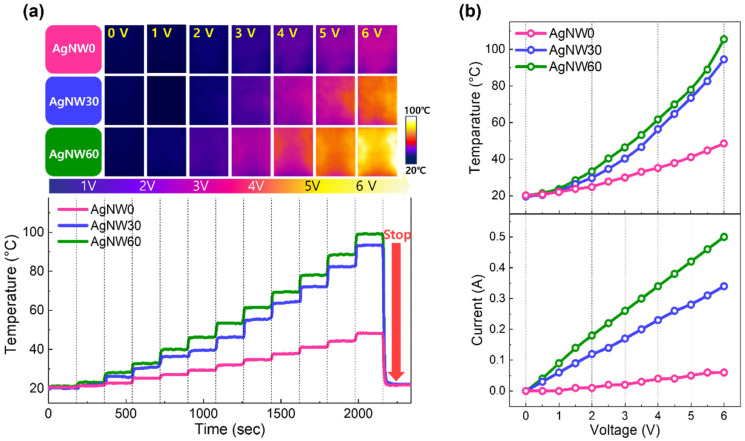
(**a**) The temperature profiles of fabrics with AgNW0, AgNW30, and AgNW60 as the voltage is increased up to 6 V in 0.5 V intervals, with each voltage step maintained for 3 min. Infrared images captured at selected voltages are displayed at the top. (**b**) A graph illustrating the relationships between temperature and voltage (**upper panel**) and between current and voltage (**lower panel**).

Figure 3a shows the Joule heating characteristics of fabrics with AgNW0, AgNW30, and AgNW60, where the heating performance was evaluated by incrementally increasing the voltage up to 6 V in 0.5 V intervals. All specimens demonstrated temperature increases upon increasing voltages and exhibited a linear I-V (Ohmic) relationship, as summarized in Figure 3b. The resistance modulation achieved by sputtering proved to be an efficient method for controlling the heat generated by the fabric. The fabric temperatures reached saturated levels within 10 s at given applied voltages, and once stable temperatures were reached, the temperature variations remained within 2%. These findings suggest that structural modulation of AgNWs through sputtering deposition is an effective approach for tailoring the heating characteristics of fabrics. This flexibility is to be contrasted with the commercially available Ag-plated nylon yarns, which must be woven into the fabric; the manufacturing process is relatively complex, and once integrated, the yarns cannot be detached or reattached. However, a limitation of the current approach is the lack of chemical bonding between the AgNWs and the fabric, which can be addressed by developing a protective adhesive layer to improve durability for commercialization.

**Figure 4:**
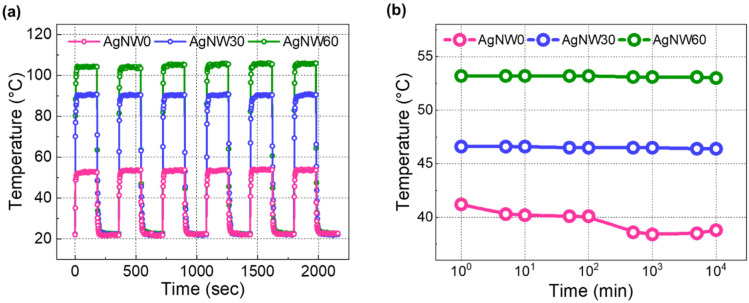
(**a**) The temperature profiles of AgNW0, AgNW30, and AgNW60 during repeated cycles of applying an external voltage (6 V) for 3 min, followed by a 3 min pause. (**b**) Temperatures for AgNW0, AgNW30, and AgNW60 while a fixed external voltage (3.5 V) is applied for a duration of 10,000 min.

Figure 4a presents the temperature profiles for AgNW0, AgNW30, and AgNW60 over six cycles of a 3 min application of external voltage supply (6 V), followed by a 3 min pause. Consistent with the results in Figure 3a, fabrics with lower resistances exhibited higher temperatures, and all fabrics displayed highly reproducible thermal profiles during the heating cycles. When voltage was applied, the temperatures for AgNW0, AgNW30, and AgNW60 reached approximately 53 °C, 90 °C, and 104 °C, respectively. Once these steady-state temperatures were reached, thermal variations were less than 2%, indicating excellent thermal stability. Figure 4b further examined the long-term heating stability of these specimens by monitoring the temperature as 3.5 V was applied continuously for 10,000 min (approximately 7 days). AgNW0 exhibited a continuous temperature drop with the increasing duration, marking an 8% decrease relative to the initial temperature. In contrast, AgNW30 and AgNW60 maintained consistent temperatures, demonstrating superior structural durability enabled by sputter deposition. This improved stability may be attributed to the smaller portion of corroded volume relative to the total Ag volume, which stunts the diffusion of corrosive agents and does not considerably increase resistance due to the reduced severity of electron surface scattering. Given the stringent requirements for thermal reproducibility and durability in heatable fabrics for commercial applications, the method presented in this study can be a promising approach.

**Figure 5:**
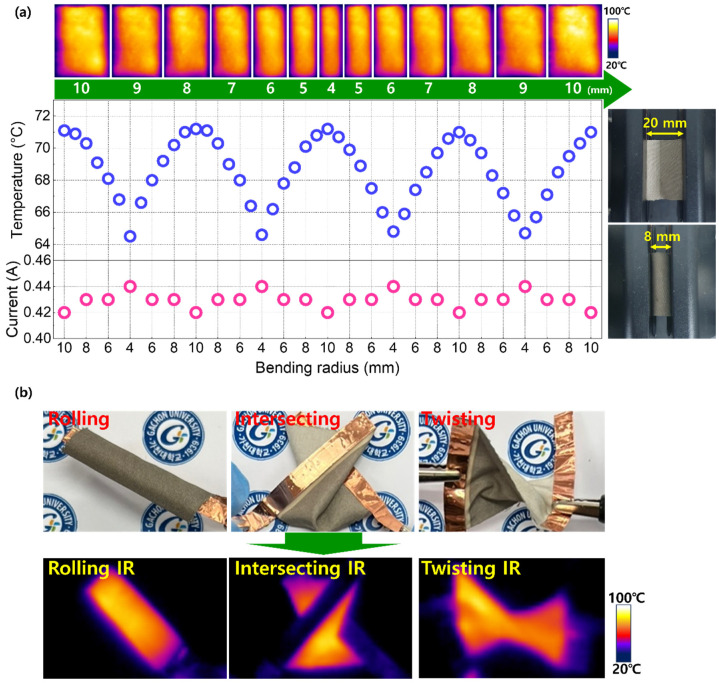
(**a**) The temperature and current profiles for AgNW60 as a function of bending radius (4–10 mm) at a fixed applied voltage of 4.5 V. IR images captured at each evaluated bending radius are presented above. (**b**) The photographs and corresponding IR images during Joule heating under complex structural deformations simulating realistic fabric conditions.

In addition to the thermal reproducibility and long-term stability examined in Figure 4, a key requirement for heatable fabrics is the capability of maintaining heating performance under highly strained conditions. Figure 5a shows the heating performance of AgNW60 during repetitive mechanical bending at a constant applied voltage of 4.5 V, along with the infrared (IR) images captured at various bending radii. While varying the bending radius from 10 to 4 mm, the fabric temperature dropped by 9.3%, along with an increase in electrical current by 4.8%. At the increased bending radius of 10 mm, however, both the fabric temperature and current reverted to their original values. This indicates that the temperature drop was due to enhanced heat dissipation associated with the increased structural deformation [38,39,40] rather than a permanent degradation in heating capability. The concurrent increase in electrical current with decreasing fabric temperatures could be attributed to reduced phonon scattering at lower temperatures. Figure 5b displays the heating performance of the fabric under complex deformation states, simulating realistic conditions for wearable electronics. The fabric demonstrated efficient heating performance in all scenarios, validating the suitability of the proposed method for producing durable and reliable heating fabrics.

## 4. Conclusions

In this study, we addressed key challenges in the development of heatable fabrics by integrating globally interconnected silver nanowires (Ag NWs) with sputter-deposited Ag atoms. By modulating the Ag NW morphology via sputtering, we achieved precise control over heating characteristics while preserving the fabric’s structural integrity and durability. Our experimental results showed that increasing Ag-ELT through sputtering significantly reduces the resistance of Ag NW-embedded fabrics, thereby enhancing heat generation efficiency. The fabrics demonstrated excellent thermal stability and reproducibility under various operating conditions, including cyclic heating and prolonged electrical stress. Further, we assessed the heating performance under complex deformation scenarios to simulate realistic conditions for wearable electronics, in which the fabrics exhibited remarkable durability. These findings suggest that our proposed method offers a promising approach for advancing heatable textile applications.

## Data Availability

The original contributions presented in the study are included in the article, further inquiries can be directed to the corresponding author.
